# Clinically Actionable Topical Strategies for Addressing the Hallmarks of Skin Aging: A Primer for Aesthetic Medicine Practitioners

**DOI:** 10.7759/cureus.52548

**Published:** 2024-01-19

**Authors:** Piercarlo Minoretti, Enzo Emanuele

**Affiliations:** 1 Occupational Medicine, Studio Minoretti, Oggiono, ITA; 2 Clinical Pathology, 2E Science, Robbio, ITA

**Keywords:** minimally invasive procedures, topical treatments, aesthetic medicine, hallmarks, aging, skin

## Abstract

In this narrative review, we sought to provide a comprehensive overview of the mechanisms underlying cutaneous senescence, framed by the twelve traditional hallmarks of aging. These include genomic instability, telomere attrition, epigenetic alterations, loss of proteostasis, impaired macroautophagy, deregulated nutrient sensing, mitochondrial dysfunction, cellular senescence, stem cell exhaustion, altered intercellular communication, chronic inflammation, and dysbiosis. We also examined how topical interventions targeting these hallmarks can be integrated with conventional aesthetic medicine techniques to enhance skin rejuvenation. The potential of combining targeted topical therapies against the aging hallmarks with minimally invasive procedures represents a significant advancement in aesthetic medicine, offering personalized and effective strategies to combat skin aging. The reviewed evidence paves the way for future advancements and underscores the transformative potential of integrating scientifically validated interventions targeted against aging hallmarks into traditional aesthetic practices.

## Introduction and background

As the body's largest organ, the skin, with its extensive surface area of 1.5-2 m2, prominently exhibits the most noticeable signs of aging [1]. These manifestations include wrinkles, thickening, coarseness, dryness, telangiectasias, reduced elasticity and firmness, irregular pigmentation, and solar lentigines [2]. Accompanying these visible changes are numerous histological alterations such as epidermal atrophy, particularly impacting the stratum spinosum, remodeling of the dermal-epidermal junction, a decrease in fibroblasts and collagen in the dermis, altered extracellular matrix (ECM) composition, and loss of subcutaneous fat [3]. These modifications, in turn, compromise the skin’s structural integrity and functionality, leading to a diminished aesthetic appeal and reduced resilience to injuries and diseases, including dermatoporosis [4] and both benign and malignant conditions [5]. In general, cutaneous aging can be viewed as a complex accumulation of inevitable and progressive phenotypic alterations, shaped by intrinsic genetic and epigenetic factors as well as extrinsic influences like exposure to ultraviolet radiation (UVR), pollutants, and other environmental stressors [6]. The current understanding of cutaneous senescence can be framed by the twelve general hallmarks of aging proposed by Lopez-Otin et al. [7], encompassing genomic instability, telomere attrition, epigenetic changes, loss of proteostasis, impaired macroautophagy, deregulated nutrient sensing, mitochondrial dysfunction, cellular senescence, stem cell exhaustion, altered intercellular communication, chronic inflammation, and dysbiosis. In a recent review, Jin et al. [8] have extensively delved into the pathophysiology of skin aging, examining it through the lens of seven key hallmarks (genomic instability, telomere attrition, epigenetic alterations, loss of proteostasis, deregulated nutrient sensing, mitochondrial damage, cellular senescence, and altered intercellular communication).

Despite significant advancements in our understanding of skin aging mechanisms, there remains a pressing need for systematic research into molecular interventions that target its primary indicators. This review aims to evaluate existing topical strategies that could potentially address these aging hallmarks and explore their integration with traditional aesthetic medicine techniques. The ultimate objective is to enhance the effectiveness of current aesthetic procedures by leveraging the current molecular knowledge of skin aging.

## Review

Topical targeting of skin aging hallmarks

Targeting Genomic Instability

As organisms age, the nuclear genome of skin cells is subject to growing instability [9], a phenomenon supported by numerous studies that have identified an increased incidence of various forms of DNA damage in aged skin compared to younger skin [10]. While it remains challenging to definitively ascertain whether this is a cause or merely a consequence of the aging process, the DNA damage theory, which posits that genomic instability significantly contributes to the progression of age-related changes, is increasingly gaining acceptance [11]. This conceptual framework is partially substantiated by cases of progeroid syndromes, such as Werner syndrome [12] and Cockayne syndrome [13], where inherited DNA repair defects result in an accelerated accumulation of DNA damage, leading to premature aging of certain organs, including the skin. Photoaging, another crucial factor contributing to genomic instability, results from chronic exposure to UVR, which not only induces genetic mutations [14] but also decreases the effectiveness of DNA repair mechanisms [15]. The various wavelengths of UVR that reach the Earth’s surface can cause different types of DNA damage [14]. Specifically, UVB radiation, which ranges from 290-320 nm, directly excites the DNA molecule, leading to the formation of cyclobutane pyrimidine dimers (CPDs). Conversely, UVA radiation, which ranges from 320-400 nm, damages DNA indirectly through photosensitized reactions [16]. This process involves the absorption of photons by non-DNA chromophores, generating reactive oxygen species (ROS) that lead to oxidative DNA damage [16]. Currently, the main clinically actionable strategy that can be used to increase genomic stability as the skin ages consists of the topical application of xenogenic DNA repair enzymes, including topical T4 endonuclease (T4N5), photolyase, endonuclease, and 8-oxoguanine glycosylase photolyase (OGG1), encapsulated in liposomes [17,18]. Photolyase, an enzyme extracted from microalgae such as *Anacystis nidulans*, is effective in repairing UVB-induced direct DNA damage [19,20]. This enzyme utilizes light in the 300 to 500 nm wavelength range to catalyze the repair of CPDs caused by UVB radiation both in vitro and in vivo [21]. Endonucleases are enzymes that can cleave phosphodiester bonds within a polynucleotide chain [22]. Certain types of endonucleases, such as T4N5 [23] and endonucleases derived from *Micrococcus luteus* [22], are recognized for their role in repairing CPDs. In addition, endonucleases are part of the base excision repair pathway, which is a key mechanism for repairing small, non-helix-distorting base lesions from the DNA [24]. Finally, OGG1, or 8-oxoguanine DNA glycosylase, is an enzyme that plays a pivotal role in repairing 8-oxo-7,8-dihydro-2’-deoxyguanosine (8OHdG), a common marker of oxidative DNA damage [25]. Interestingly, the activity of OGG1 has been observed to decline with age, leading to an accumulation of 8OHdG and associated mitochondrial dysfunctions [26]. In summary, the topical application of DNA repair enzymes encapsulated within liposomes offers a promising actionable intervention to preserve genomic integrity in skin cells. By directly reversing the accumulation of DNA damage over time, a key driver of cutaneous aging, this approach may mitigate age-related skin changes.

Targeting Telomere Attrition

Telomeres serve as protective caps at the extremities of linear chromosomes, playing a crucial role in safeguarding the genome's stability [27]. With each cell division, telomeres become progressively shorter, ultimately leading to their loss and a state of irreversible cell cycle arrest [28]. Current research is exploring various strategies to address telomere attrition. One experimental strategy leverages the telomere shelterin protein, specifically the telomeric repeat binding factor 2 (TRF2), which safeguards telomeres from DNA damage [29]. Given that TRF2 inhibition can expedite telomere shortening and DNA damage, restoring the compromised telomeric shelterin machinery could provide a potential approach to mitigate telomere attrition [30]. Another preclinical strategy involves therapeutically targeting the reverse transcriptase telomerase enzyme, which is responsible for creating new telomeric DNA from an RNA template [31]. However, its activity is typically limited to cells that require a high proliferation capacity (e.g., stem cells) [32]. Unfortunately, these strategies are still in the experimental phase, and their application is confined to preclinical settings. Building on the understanding that the skin has developed intricate DNA repair mechanisms to counteract telomere degradation, we have previously explored the effectiveness of photolyase from *Anacystis nidulans* and endonuclease from *Micrococcus luteus* in preventing telomere attrition in skin cells caused by experimental UVR irradiations [22]. Notably, these xenogenic DNA repair enzymes elicited a significant reduction in the rate of telomere shortening compared to a negative control [22]. As a result, the topical application of these enzymes could represent a clinically actionable approach for addressing not only age-related genomic instability but also cutaneous telomere attrition.

Targeting Epigenetics Alterations

Epigenetic modifications, which include changes in DNA methylation, histone alterations, and the expression of non-coding RNAs, are recognized as crucial mechanisms in regulating gene expression during skin aging [33]. While these epigenetic shifts do not alter the DNA sequence itself, they can lead to the activation or repression of specific genes by influencing the transcription process, which is the first step in gene expression [34]. This regulation can alter the levels of messenger RNA produced, which are then translated into polypeptide chains during protein synthesis [33]. Epigenetic skincare is an emerging field that focuses on developing topical ingredients that can alter gene expression for skin enhancement [34]. One such ingredient is equol, an isoflavone derived from the soy isoflavone daidzein by gut bacteria [35]. When equol is applied to the skin for eight weeks, it can reduce the average level of long interspersed element-1 (LINE-1), a marker of global DNA methylation [35]. Notably, this decrease in LINE-1 methylation was paralleled by a decrease in telomere attrition at the molecular level and noticeable improvements in skin roughness, texture, and smoothness from an aesthetic perspective [35]. Other actionable epigenetic skincare ingredients include sulforaphane from cauliflower [36], anacardic acid from *Anacardium occidentale* (cashew) nutshells [37], epigallocatechin-3-gallate from green tea [38], and palmitoyl-KVK-L-ascorbic acid conjugate [39]. These compounds exert their effects by decreasing the activity of DNA methyltransferase 1 (DNMT1), the most abundant enzyme involved in DNA methylation. By inhibiting DNMT1, these actives can increase the expression of procollagen, a key protein involved in maintaining skin elasticity and strength [34,39].

Targeting Loss of Proteostasis

The disruption of protein quality control, or proteostasis, leading to the accumulation of oxidized proteins (i.e., protein carbonylation), is a hallmark of both chronological and photo-induced aging in the skin [40]. Given that this phenomenon occurs in dermal fibroblasts during both intrinsic aging and following UVR exposure, an altered proteome has been recently proposed by Benoit et al. [41] as a central mechanism underlying skin aging. Interestingly, an extract derived from the bacterium *Arthrobacter agilis* has been identified as a potential novel agent for proteome protection due to its chaperone-like properties [42]. Additionally, this extract has shown the ability to protect the skin’s proteome from carbonylation, a process that can lead to protein misfolding and degradation, thereby suggesting its potential for anti-aging applications [41,42]. Glycation, a specific type of protein damage where a sugar molecule interacts with a protein to form advanced glycation end products (AGEs), is another significant contributor to age-related loss of skin proteostasis [43]. Typically, the rate of protein turnover within cells is rapid enough to prevent the accumulation of AGEs. However, some extracellular proteins, such as collagen, can have a turnover rate of up to a decade. Over time, AGEs form cross-links between elastin and collagen proteins, leading to age-related loss of skin elasticity and firmness [44]. Individuals with type 2 diabetes exemplify the extreme effects of glycation, as their inability to efficiently regulate plasma glucose levels results in a significantly accelerated rate of glycation [45]. Several skincare ingredients have been identified as potentially effective in reducing protein glycation and AGEs formation, including Akebia quinata extract [46], carnosine [47], and resveratrol derivatives [48]. Unfortunately, while these ingredients can potentially mitigate the effects of glycation, they cannot reverse the process entirely.

Targeting Disabled Macroautophagy

Macroautophagy, often referred to simply as autophagy, is a primarily degradative pathway that occurs in all eukaryotic cells [49]. It is used for recycling cytoplasm to generate macromolecular building blocks and energy under various conditions, to remove superfluous and damaged organelles, and to prevent the accumulation of toxic and misfolded proteins. Autophagy is continuously active in the outer skin epithelium during the cornification of keratinocytes, enhancing resistance to environmental stress [50]. While rapidly renewing epidermal epithelium can tolerate experimental autophagy suppression in stress-free conditions, long-lasting skin cells like Merkel cells, melanocytes, and sweat gland secretory cells rely on autophagy for cellular balance and proper function during aging [51]. Mounting evidence indicates that autophagy plays a vital role in skin-related processes that affect aesthetic appearance, such as the functioning of sebaceous glands, angiogenesis, and melanogenesis [52]. Consequently, stimulating autophagy in the skin has emerged as a key strategy for enhancing its aesthetic appeal. Unfortunately, despite the abundance of preclinical autophagy activators, only a limited number have undergone topical testing in humans. Lee and colleagues developed a peptide that activates autophagy and demonstrated that it reduced skin surface lipids and trans-epidermal water loss in acne-prone skin [53]. In a separate study, an autophagy activator termed heptasodium hexacarboxymethyl dipeptide-12 (Aquatide™) induced a statistically significant increase in skin elasticity after four and eight weeks of use [54]. The Aquatide™-treated group also showed a significant reduction in carbonylated proteins in the stratum corneum [54]. A phytocosmetic formulation containing extracts from* Myrothamnus flabellifolia* leaf and *Coffea arabica *seed (MflCas), which can induce autophagy, was shown to improve various skin features in volunteers, particularly signs of skin aging and pigmentation [55]. After 56 days of treatment with 2% MflCas, there was a reduction in spot length, skin contrast, and an increase in skin homogeneity and skin lightening effect on the hand skin [55]. On the face, the treatment reduced spot length, wrinkle area, and wrinkle volume while increasing face skin homogeneity [55]. In a separate study, a topical skincare serum termed A+ was developed using human fibroblast-conditioned media and was found to upregulate genes associated with autophagy [56]. Clinical assessments showed that the A+ serum provided significantly greater reductions in coarse wrinkles, fine wrinkles, sagging, and overall hyperpigmentation compared to the placebo. Subjects reported appearing younger, with a median decrease in self-perceived age of six years after 12 weeks of use [56]. Recent findings also suggested that the topical application of magnetized saline water, when incorporated into a serum, can enhance skin biophysical parameters in women seeking to improve the appearance of their facial and neck skin [57]. Intriguingly, these enhancements were linked to the activation of cutaneous autophagy [57].

Targeting Deregulated Nutrient-Sensing

The dysregulation of nutrient-sensing pathways [58], including sirtuins, insulin-like growth factor 1 (IGF-1), the mechanistic target of rapamycin (mTOR), and AMP-activated protein kinase (AMPK), plays a significant role in the process of skin aging. Sirtuins are a family of signaling proteins that play a crucial role in regulating metabolism. They are particularly activated in states of calorie restriction and in the presence of the coenzyme nicotinamide adenine dinucleotide (NAD) [59]. Nicotinamide, a water-soluble form of vitamin B3, when applied topically, has been shown to induce the expression of sirtuins, replenish cellular NAD levels, and enhance mitochondrial energetic [60]. From a clinical perspective, this leads to an improvement in the extracellular matrix and skin barrier, and a reduction in the skin pigmentation process [60]. Topical creams containing capsaicin have been demonstrated to stimulate the production of IGF-1 through the activation of sensory neurons [61]. A clinical study involving the application of 0.01% capsaicin cream to the faces of 17 healthy female volunteers over seven days resulted in a significant enhancement of cheek skin elasticity [61]. These findings suggest that capsaicin could be an actionable ingredient for combating age-related morphological changes in the skin by elevating dermal IGF-1 levels. In a distinct exploratory, placebo-controlled, interventional trial, rapamycin, an FDA-approved drug that targets the mTOR complex, has been demonstrated to enhance both the appearance and histology of skin in numerous participants [62]. Furthermore, metformin, a powerful AMPK activator and the most frequently prescribed oral medication for diabetes has been consistently proposed for topical repurposing in anti-aging medicine [63]. Intriguingly, the rejuvenating effects of locally applied metformin have been extensively reported in preclinical studies [64,65].

Targeting Mitochondrial Dysfunction

Skin aging is marked by a significant rise in mutations and deletions in mitochondrial DNA [66]. This accumulation is in turn linked to a steady decrease in mitochondrial function and an increase in the production of ROS. The resulting oxidative stress in keratinocytes, coupled with a concurrent reduction in mitochondrial membrane potential, promotes an unfavorable metabolic shift from oxidative phosphorylation to anaerobic glycolysis [67]. Furthermore, youthful skin is distinctly characterized by a well-connected network of mitochondria within the keratinocytes. In contrast, aged skin exhibits a substantial reduction in mitochondrial clusters, with a notably fragmented mitochondrial network [66,67]. This fragmentation suggests inefficient recycling and excessive mitophagy, the selective autophagy of mitochondria [67]. Anti-aging skincare strategies have focused on combating mitochondrial dysfunction by creating a wide array of antioxidants to neutralize ROS [68]. Additionally, several compounds, such as resveratrol, epicatechins, curcumin, and phytoestrogens, have been identified for their ability to enhance mitochondrial biogenesis when applied topically [69]. However, definitive clinical evidence from human studies remains to be established. Conversely, it is noteworthy that triclosan, a common antimicrobial ingredient in many consumer and healthcare products, has been implicated in causing skin mitochondrial dysfunction [70]. This is evidenced by its impact on mitochondrial ROS production, membrane potential, and morphology, which may inadvertently contribute to the acceleration of cutaneous aging [70].

Skin Aging and Cellular Senescence

Cellular senescence refers to the process where mitotic cells age, leading to a permanent state of growth arrest while remaining metabolically active [71]. This condition is marked by the cell loss of proliferative capacity, resistance to apoptosis, and the release of pro-inflammatory and tissue-degrading molecules, collectively known as the senescence-associated secretory phenotype (SASP) [72]. The exact role of senescent cells in skin aging, whether they are a primary cause or a result that further intensifies the aging process, is still being actively researched [73]. Recent advancements have led to the identification of specific compounds, known as senolytics, which are designed to selectively eliminate senescent cells, and senomorphics, which aim to mitigate the SASP [74]. While there is increasing preclinical and experimental evidence supporting the potential effectiveness of senotherapeutics in reducing the burden of skin senescence, in vivo interventions using senolytics and senomorphics in humans are still limited [75]. The protein p16INK4A, a cyclin-dependent kinase inhibitor and tumor suppressor, is a known biomarker of cellular senescence [76]. A clinical study by Chung et al. [62] demonstrated that the topical application of rapamycin, an mTOR inhibitor, decreased the skin expression of the p16INK4A protein, suggesting a reduction in cellular senescence in subjects over the age of 40 who showed signs of age-related photoaging and loss of dermal volume. The antidiabetic medication metformin, along with certain natural compound derivatives, specifically apigenin and kaempferol, have been shown to significantly reduce the production of the SASP in senescent fibroblasts induced by bleomycin [77]. However, these compounds should be rigorously tested in vivo to evaluate their ability to decrease senescence markers in clinical trials.

Targeting Stem Cell Exhaustion

Stem cells, characterized by their pluripotency and self-replication capabilities, can differentiate into various functional cells under specific conditions [78]. They play a crucial role in tissue repair and the maintenance of tissue homeostasis. In the context of skin health, the decline in stem cell populations is closely associated with skin atrophy, fragility, and changes in pigmentation [79]. While the self-renewal capacity of stem cells tends to decrease with skin aging, it is observed to increase during wound repair [80]. Amniotic membrane stem cells (AMSCs), adipose-derived stem cells (ADSCs), and human umbilical cord mesenchymal stem cells (hUC-MSCs) are all being explored for their potential in skin rejuvenation due to their regenerative properties [81]. AMSCs are multipotent with low immunogenicity and can be easily obtained from the placenta, avoiding ethical issues associated with embryonic stem cells [82]. Their anti-aging potential has been associated with the release of growth factors, angio-modulatory cytokines, anti-bacterial peptides, and anti-inflammatory molecules [82]. A study showed that a topical application of AMSC metabolic products combined with vitamin E significantly improved wrinkles, ultraviolet spots, and pores in patients with photoaging [83]. ADSCs have also shown promise for facial skin rejuvenation [84]. When injected into the dermis, ADSCs improve skin density, hydration, and the number of capillary vessels. However, the survival of the graft and the outcomes depend on various factors, including patient age and the technique used for fat tissue harvesting and graft injection [84]. A separate study demonstrated that hUC-MSCs-conditioned media combined with microneedling resulted in statistically better effects on skin brightness and texture compared to microneedling alone [85]. Currently, other types of stem cells, including bone marrow stem cells and human induced pluripotent stem cells, are being explored in preclinical models but are not yet actionable in the clinical setting [86,87].

Targeting Altered Intercellular Communication

Intercellular communication is a critical process that enables cells to transmit and receive messages, playing a vital role in maintaining tissue homeostasis and function [88]. In the context of skin aging, this communication can be disrupted, leading to a decline in skin integrity and function [8]. In the realm of skin aging, exosomes, defined as small vesicles that transport proteins, lipids, and nucleic acids between cells, have garnered significant interest [89,90]. Studies have explored the use of exosomes derived from various sources, such as bovine milk or human placental mesenchymal stem cells, as a means to deliver beneficial molecules to the skin [90]. This could potentially enhance moisture retention, stimulate collagen production, and boost skin repair [89,90]. Another area of focus is gap junctions, which enable direct cytoplasmic connections between neighboring cells, facilitating the exchange of ions, metabolites, and signaling molecules [91]. As age progresses, the expression and functionality of connexins, the proteins forming gap junctions, may alter, leading to compromised cellular communication and tissue function [92]. A growth factor-based skincare serum, known as A+, developed using human fibroblast-conditioned media, has recently been shown to act on this altered intercellular communication through gap junctions [56]. Notably, saccharide isomerate, a key component of this product derived from marine exopolysaccharides, has been demonstrated to facilitate direct intercellular communication [56].

Targeting Chronic Inflammation

Inflammaging is characterized as a persistent, low-level inflammation that is non-infectious and gradually develops as a natural component of the aging process [93]. In the context of cutaneous aging, inflammaging is influenced by epidermal dysfunction, which leads to a local and systemic increase in pro-inflammatory cytokines [94]. Moreover, in the aging epidermis, there is a diminished presence of Langerhans cells, and their responsiveness to migrate towards sites of injury or infection is reduced [95]. In older individuals, senescent keratinocytes are more prevalent, and they secrete greater amounts of proinflammatory interleukin-1α compared to younger individuals [94,95]. Melanocytes in older subjects also exhibit increased signs of inflammaging [95]. Additionally, as individuals age, the skin surface pH tends to increase significantly [96]. This elevated pH not only destabilizes the epidermal permeability barrier but also boosts the activity of kallikreins, enzymes that can degrade the epidermal structure and activate protease-activated receptor 2, potentially leading to inflammation and itching [97]. Consequently, skin inflammaging is often accompanied by persistent pruritus, especially in low-humidity environments [98]. This itching can prompt scratching, which further impairs the epidermal barrier and can exacerbate inflammation, creating a vicious cycle [99]. While many anti-inflammatory compounds have demonstrated potential for alleviating cutaneous inflammation when applied topically, comprehensive studies examining their effects on inflammaging are still limited. In a preliminary investigation, Yap demonstrated that a nanoemulsion formulation containing a tocotrienol-rich fraction effectively reduced experimental signs of inflammaging induced by exposure to UVR [100]. However, the actionability of this approach in mitigating age-related inflammaging still requires validation through long-term clinical trials.

Targeting Dysbiosis

Several studies have observed alterations in the diversity of the skin microbiome with age. Generally, older skin has been reported to exhibit higher alpha diversity compared to younger skin [101]. This increased microbial diversity on aged skin may have implications for skin physiological integrity, function, and susceptibility to external factors [102]. Research from Japan has drawn comparisons between the bacterial species found in younger adults (aged 21-37 years) and those in older adults (aged 60-76 years) [103]. The findings indicated a notable rise in *Corynebacterium* on the cheeks and forehead and *Acinetobacter *on the scalp in the older demographic. On the other hand, there was a decline in *Cutibacterium *on the cheeks, forehead, and forearms in the same group [103]. Additional research has linked aging with a heightened presence of corynebacterial taxa, including *Corynebacterium kroppenstedtii *and *Corynebacterium amycolatum*, especially on the forehead [104]. Another investigation involving 30 healthy Thai females aged 19-57 years reported Firmicutes as the most prevalent bacterium in both healthy older adults and young adults prone to acne [105]. Given this evidence, the skincare industry is increasingly focusing on topical products that may combat skin aging through the inclusion of topical prebiotics, probiotics, or microbiome-targeted ingredients [106,107]. Another actionable approach for targeting skin dysbiosis involves the use of bacteriocins, which are antimicrobial peptides produced by bacteria [108]. When applied topically, bacteriocins can help reduce the colonization of proinflammatory bacterial species, such as *Staphylococcus aureus*, thereby promoting a healthier skin microbiome [108]. Table 1 summarizes the main topical interventions with the potential to address the 12 hallmarks of skin aging.

**Table 1:** Topical actionable interventions for skin aging hallmarks *Indicates clinically tested topicals which are not routinely available or approved for cosmetic use.

Skin aging hallmark	Topical actionable intervention
Genomic instability	Photolyase, endonuclease, 8-oxoguanine DNA glycosylase
Telomere attrition	Photolyase, endonuclease, equol
Epigenetics alterations	Equol, sulforaphane, anacardic acid, epigallocatechin-3-gallate, palmitoyl-KVK-L-ascorbic acid conjugate
Loss of proteostasis	Arthrobacter agilis extract, Akebia quinata extract, carnosine, resveratrol derivatives
Impaired macroautophagy	Aquatide™, Myrothamnus flabellifolia leaf and Coffea arabica seed extracts, A+ skin serum^®^, magnetized water
Deregulated nutrient sensing	Nicotinamide, capsaicin, topical rapamycin*, topical metformin*
Mitochondrial dysfunction	Resveratrol, epicatechins, curcumin, phytoestrogens
Cellular senescence	Topical rapamycin*
Stem cell exhaustion	Amniotic membrane stem cells, adipose-derived stem cells
Altered intercellular communication	A+ skin serum^®^, saccharide isomerate
Chronic inflammation	Nanoemulsions containing tocotrienol-rich fractions
Dysbiosis	Prebiotics, probiotics, microbiome-targeted ingredients, bacteriocins

Non-surgical procedures in aesthetic medicine in relation to hallmarks of skin aging

Botulinum Neurotoxin Type A (BoNT/A) Injections

Botulinum neurotoxin type A injections have become the most popular non-surgical aesthetic procedure globally due to their effectiveness and high patient satisfaction [109]. Their application has expanded from the upper face to include the midface, lower face, and neck [110]. The Global Aesthetics Consensus Group has advocated for a nuanced approach to BoNT/A use, favoring neuromodulation over complete muscle paralysis [111]. This approach includes prescribing lower doses for the upper face, combining treatments with hyaluronic acid fillers more frequently, and utilizing intradermal injections to fine-tune the toxin’s impact when necessary [111]. Interestingly, experimental and preclinical studies suggest that BoNT/A may paradoxically contribute to skin aging. Research by Fooladvand et al. [112] using a Drosophila melanogaster model revealed that BoNT/A can cause DNA damage, activate caspase-3 and -9, and induce cellular senescence, negatively affecting at least three aging hallmarks (i.e., genomic instability, loss of proteostasis, and cellular senescence). In addition, Miao et al. [113] found that BoNT/A treatment in human dermal fibroblasts altered the expression of long non-coding RNAs, with an increase in FOS expression linked to cellular senescence. Finally, Salari et al. [114] observed that individuals with pre-existing mitochondrial dysfunction might experience exacerbated muscle atrophy following BoNT/A injections and suggested that the toxin could impair myogenic proliferation and reduce satellite cells’ self-renewal capacity. Collectively, these findings indicate that BoNT/A could potentially impact six aging hallmarks: genomic instability, loss of proteostasis, cellular senescence, epigenetic alterations, mitochondrial dysfunction, and stem cell exhaustion. This highlights the importance of further investigation into the long-term effects of BoNT/A injections on the aging process of the skin. Moreover, it would be beneficial to explore the integration of BoNT/A injections with topical, actionable strategies aimed at addressing the six aging hallmarks that could be potentially affected by this neurotoxin.

Dermal Fillers

Injectable dermal fillers, ranking as the second most common non-invasive aesthetic procedure, are increasingly utilized for soft tissue augmentation and rejuvenation [115]. These fillers serve to restore volume to skin depressions, thereby contributing to a youthful and rejuvenated appearance [116]. The market currently offers a growing variety of these fillers, with common biodegradable examples including hyaluronic acid, calcium hydroxylapatite, poly-L-lactic acid, and dextran beads in hyaluronic acid [115,116]. While the primary mechanism of action of dermal fillers is to provide sufficient physical volume to compensate for loss, there is also evidence suggesting that these fillers are not inert. They may exert bio-stimulatory effects, increasing fibroblast activity and stimulating collagen synthesis [117], which ultimately affects the quality of subcutaneous tissue. Despite the lack of systematic studies investigating the effects of dermal fillers on the hallmarks of skin aging, it is important to note that the most common chemical cross-linker used in hyaluronic acid-based dermal fillers, 1,4-butanediol diglycidyl ether (BDDE), may potentially induce oxidative DNA damage in the form of 8OHdG [118]. The feasibility of incorporating a DNA repair enzyme like OGG1 to mitigate potential BDDE-induced genomic instability warrants further investigation.

Chemical Peels

Chemical peeling, or chemical exfoliation, involves applying acidic solutions like salicylic, lactic, and glycolic acids to the skin to promote exfoliation, depigmentation, and revitalization [119]. There are three levels of chemical peels: superficial, medium, and deep, chosen based on the extent of skin damage and patient expectations [120]. Superficial peels remove the top layer of the epidermis, leading to rapid skin regeneration, spot reduction, and an improved appearance of fine lines and wrinkles by promoting fibroblast activity and fiber regeneration. Medium peels target more damaged skin, penetrating deeper to improve the middle layers of the skin. They can treat fine wrinkles and discolorations, making the skin appear smoother and more youthful. However, they may cause redness and peeling for up to two weeks post-treatment. Deep peels are aggressive treatments for severe wrinkles and scars [119,120]. In relation to the effects of chemical peeling on the hallmarks of skin aging, Kornhauser and colleagues [121] found that glycolic acid treatment, unlike salicylic acid, led to heightened sensitivity of human skin to solar simulated radiation. This outcome was associated with DNA damage [121]. Given the importance of post-peeling care [119], it is recommended to conduct clinical tests on topical DNA repair enzymes. Intriguingly, in vitro research suggests that lactic acid may shield skin fibroblasts from aging-related mitochondrial dysfunction [122]. However, it remains to be determined if this protective effect against a key hallmark of aging is also present in vivo during chemical peeling treatments.

Microdermabrasion

Microdermabrasion is a non-chemical procedure that superficially resurfaces the skin by removing the stratum corneum [123]. This technique utilizes a vacuum system to propel abrasive crystals against the skin, which gently exfoliates the surface, aiding in dermal remodeling [124]. While there is no definitive evidence linking microdermabrasion to direct effects on the hallmarks of skin aging, this technique shows potential for enhancing the skin’s absorption and increasing the effectiveness of targeted anti-aging treatments [125]. Accordingly, research suggests that the permeability of skin treated with microdermabrasion can be significantly higher compared to untreated skin [126], which may be beneficial for the delivery of topical compounds targeted against the hallmarks of aging. Hence, the application of this method could be valuable for clinical applications in improving the delivery of targeted anti-aging therapies.

Laser-Based Techniques

Laser techniques are commonly employed in skin rejuvenation and resurfacing procedures [127]. Ablative lasers work by vaporizing the outermost skin layer, the stratum corneum, down to the dermis [128]. In contrast, nonablative fractional lasers create conical zones of coagulation in the epidermis and upper dermis [128]. Fractional picosecond lasers generate intraepidermal and/or dermal vacuoles via laser-induced optical breakdown. Traditional ablative lasers cause widespread vaporization, while fractional ablative lasers form columns of tissue ablation [128]. These laser procedures can influence various aspects of skin aging, both beneficially and detrimentally. During irradiation, mitochondria are the primary targets, and laser photons can help fight mitochondrial dysfunction [129]. Research has also shown that fractionated laser resurfacing could increase dermal IGF-1 levels, thereby addressing deregulated nutrient sensing [130]. Furthermore, transient photoactivation of epidermal stem cells by femtosecond laser has been observed [131]. However, there are potential negative impacts as well. For instance, Morkunas et al. [132] reported a possible genotoxic effect of new-generation 206 nm femtosecond solid-state laser irradiation, primarily in the form of CPDs. If this potentially harmful effect is confirmed in human studies, it could be mitigated by applying topical DNA repair enzymes after laser treatment.

Platelet-Rich Plasma (PRP)

Platelet-rich plasma is a self-derived plasma preparation with a high concentration of platelets [133]. Being abundant in growth factors such as platelet-derived growth factor, transforming growth factor, vascular endothelial growth factor, and IGF-1 [134], PRP is extensively used in anti-aging medicine due to its potential to boost skin regeneration [135]. Theoretically, PRP’s role in tissue repair and regeneration could impact aging hallmarks like cellular senescence and stem cell exhaustion. A recent innovation, the Qigeneration Procedure, employs the QiLaser to activate very small embryonic-like pluripotent stem cells in autologous PRP [136]. This technology is currently being investigated for its potential anti-aging applications, particularly its influence on epigenetic changes. Table 2 summarizes the effects of non-surgical aesthetic medicine procedures on skin aging hallmarks and potential solutions.

**Table 2:** Effects of non-surgical aesthetic medicine procedures on skin aging hallmarks and potential solutions

Non-surgical aesthetic medicine procedure	Effects on skin aging hallmarks	Potential solutions
Botulinum neurotoxin type A injections	Negative impact on at least six aging hallmarks (genomic instability, loss of proteostasis, cellular senescence, epigenetic alterations, mitochondrial dysfunction, and stem cell exhaustion)	Integrate topical treatments aimed at combating the affected aging hallmarks in all patients treated with botulinum neurotoxin type A injections
Dermal fillers	1,4-butanediol diglycidyl ether (BDDE) used in hyaluronic acid-based fillers may negatively affect genomic instability	Incorporating a DNA repair enzyme like 8-oxoguanine glycosylase photolyase in filler formulations should be explored
Chemical peels	Glycolic acid may potentially induce genomic instability	Use topical DNA repair enzymes in post-peeling care
Microdermabrasion	No known effects	-
Laser-based techniques	The new-generation 206 nm femtosecond solid-state laser irradiation may potentially induce genomic instability	Use topical DNA repair enzymes in post-laser care
Platelet-rich plasma	Positive impact on at least two aging hallmarks (cellular senescence and stem cell exhaustion)	-

## Conclusions

In this review, we conducted an extensive analysis of the state-of-the-art topical interventions designed to tackle the 12 hallmarks of skin aging. We evaluated not only the individual effectiveness of these cutting-edge strategies but also their potential synergistic effects when paired with leading non-surgical procedures in aesthetic medicine. The integration of targeted topical therapies with minimally invasive techniques could mark substantial progress in aesthetic medicine, providing tailored, potent solutions for mitigating skin aging. The reviewed evidence paves the way for future advancements and underscores the transformative potential of integrating scientifically validated interventions targeted against aging hallmarks into traditional aesthetic practices.
